# Mighty Dwarfs: *Arabidopsis* Autoimmune Mutants and Their Usages in Genetic Dissection of Plant Immunity

**DOI:** 10.3389/fpls.2016.01717

**Published:** 2016-11-17

**Authors:** Rowan van Wersch, Xin Li, Yuelin Zhang

**Affiliations:** ^1^Department of Botany, University of British Columbia, VancouverBC, Canada; ^2^The Michael Smith Laboratories, University of British Columbia, VancouverBC, Canada

**Keywords:** *Arabidopsis*, autoimmunity, suppressor screening, PAMP-triggered immunity (PTI), effector-triggered immunity (ETI), receptor-like kinases, NB-LRR proteins

## Abstract

Plants lack the adaptive immune system possessed by mammals. Instead they rely on innate immunity to defend against pathogen attacks. Genomes of higher plants encode a large number of plant immune receptors belonging to different protein families, which are involved in the detection of pathogens and activation of downstream defense pathways. Plant immunity is tightly controlled to avoid activation of defense responses in the absence of pathogens, as failure to do so can lead to autoimmunity that compromises plant growth and development. Many autoimmune mutants have been reported, most of which are associated with dwarfism and often spontaneous cell death. In this review, we summarize previously reported *Arabidopsis* autoimmune mutants, categorizing them based on their functional groups. We also discuss how their obvious morphological phenotypes make them ideal tools for epistatic analysis and suppressor screens, and summarize genetic screens that have been carried out in various autoimmune mutant backgrounds.

## Introduction

The ability to detect and respond to pathogens is essential for the survival of multicellular organisms. Plants have developed sophisticated immune systems to combat microbial pathogens. Recognition of Pathogen-Associated Molecular Patterns (PAMPs), components important for pathogen lifestyles such as bacterial flagellin, by Pattern Recognition Receptors (PRRs) leads to activation of PAMP-triggered immunity (PTI) (Boller and Felix, 2009). Most PRRs belong to the transmembrane receptor-like kinase (RLK) or receptor-like protein (RLP) families (Monaghan and Zipfel, 2012). Unlike RLKs, which contain extracellular, transmembrane, and intracellular kinase domains, RLPs lack the intracellular kinase domain.

In response to pathogens that deliver effector proteins to promote virulence by avoiding detection by the host cells or inhibiting PTI, plants have evolved resistance (R) proteins, which detect effectors and trigger an immune response termed effector triggered immunity (ETI). R proteins recognize effectors either by directly binding to the effectors or indirectly sensing modifications to host proteins caused by the effectors (Jones and Dangl, 2006). Most R proteins contain a central nucleotide-binding domain (NB) and C-terminal leucine-rich repeats (LRRs), with either a Toll-interleukin 1-like receptor (TIR) or a coiled-coil (CC) domain at their N termini.

Plant immunity is under tight negative control to avoid activation in the absence of pathogens. Immune receptors are generally maintained at inactive states and activated only upon detection of pathogens. Loss-of-function mutations in negative regulators and gain-of-function mutations in plant immune receptors often lead to autoimmunity. The typical phenotypes of autoimmune mutants include dwarfism, elevated salicylic acid levels, constitutive expression of defense genes and enhanced disease resistance to pathogens, and in some cases also spontaneous lesion formation. They have been instrumental in assisting our studies of plant immunity. Previous reviews have highlighted lesion mimic mutants (Bruggeman et al., 2015; Rodriguez et al., 2016). Here we are focusing on *Arabidopsis* autoimmune mutants (**Table 1**) and their application in studying plant immunity.

**Table 1:** *Arabidopsis thaliana* autoimmune mutants organized by protein class.

Mutant	Protein class	Reference
*snc1^∗^*	TIR-NB-LRR	Li et al., 2001; Zhang et al., 2003
*ssi4^∗^*	TIR-NB-LRR	Shirano et al., 2002
*slh1^∗^*	TIR-NB-LRR	Noutoshi et al., 2005
*chs3-2D, chs3-1^∗^*	TIR-NB-LRR	Yang et al., 2010; Bi et al., 2011
*chs2^∗^*	TIR-NB-LRR	Huang et al., 2010
*uni-1D^∗^*	CC-NB-LRR	Igari et al., 2008
*bak1 bkk1*	LRR-RLKs	He et al., 2007
*bir1*	LRR-RLK	Gao et al., 2009
*snc4-1D^∗^*	RLK	Bi et al., 2010
*cerk1-4^∗^*	LysM-RLK	Petutschnig et al., 2014
*snc2-1D^∗^*	RLP	Zhang et al., 2010
*rin4*	Unknown	Mackey et al., 2002
*cpr1*	F-Box	Bowling et al., 1994; Gou et al., 2012
*srfr1*	TPR	Li et al., 2010a; Kim et al., 2010
*cpn1/bon1*	Copine	Hua et al., 2001; Jambunathan et al., 2001
*mkp1*	MAPK phosphotase	Bartels et al., 2009
*lsd1*	Zinc finger	Dietrich et al., 1994; Dietrich et al., 1997
*acd11*	Sphingosine transfer	Brodersen et al., 2002
*cpr5*	Nucleoporin	Bowling et al., 1997
*mekk1*	MAPKKK	Ichimura et al., 2006; Nakagami et al., 2006
*mpk4*	MAPK	Petersen et al., 2000
*mkk1 mkk2*	MAPKKs	Gao et al., 2008; Qiu et al., 2008
*acd6^∗^*	TM-ANK	Rate et al., 1999; Lu et al., 2003
*bda1-17^∗^*	TM-ANK	Yang et al., 2012
*dnd1*	CNG Ion Channel	Yu et al., 1998
*dnd2/hlm1*	CNG Ion Channel	Balagué et al., 2003; Jurkowski et al., 2004
*cpr22^∗^*	CNG Ion Channel	Yoshioka et al., 2001
*npr3 npr4*	BTB-ANK	Zhang et al., 2006
*sr1/camta3*	Transcription factor	Galon et al., 2008; Du et al., 2009
*pub13*	U-box	Li et al., 2012
*cpr6-1^∗^*	Unknown	Clarke et al., 1998
*ssi2*	S-ACP-DES	Shah et al., 2001
*syp121 syp122*	Syntaxin	Zhang et al., 2007
*cad1*	MACPF	Morita-Yamamuro et al., 2005
*nsl1*	MACPF	Noutoshi et al., 2006

*^∗^Gain of Function mutations TM, Transmembrane; ANK, Ankyrin repeat; TPR, Tetratrico-peptide repeat; S-ACP-DES, Stearoyl-acyl carrier desaturase protein.*

## Autoimmunity Caused By Gain-Of-Function Mutations in NB-LRR Proteins

Autoimmunity in a number of *Arabidopsis* mutants is caused by mutations in TIR-NB-LRR proteins. Among them, *suppressor of npr1-1, constitutive* (*snc*) *1* is one of the most well studied (Li et al., 2001). A single amino acid change in the linker region between the NB and LRR domains leads to over-accumulation of the SNC1 protein and activation of defense responses (Zhang et al., 2003). *snc1* exhibits typical autoimmune phenotypes, but does not have spontaneous cell death. Both SA-dependent and SA-independent defense pathways contribute to the enhanced pathogen resistance in *snc1*. Unlike *snc1, suppressor of salicylic acid insensitive* (*ssi*) *4* is another gain-of-function mutant of a TIR-NB-LRR protein that displays both dwarfism and spontaneous cell death (Shirano et al., 2002).

The autoimmune phenotypes in *sensitive to low humidity* (*slh)1* (an allele of *RRS1*) and *chilling sensitive* (*chs*) *3-2D* are caused by mutations in atypical TIR-NB-LRR proteins (Noutoshi et al., 2005; Bi et al., 2011). RESISTANT TO RALSTONIA SOLANACEARUM 1 (RRS1) and CHS3 contain extra C-terminal domains proposed to function as integrated decoys for pathogen effectors. RRS1 has a WRKY DNA-binding domain at its C-terminus. In *slh1*, a single amino acid insertion in the DNA-binding domain causes activation of defense responses (Noutoshi et al., 2005). CHS3 contains a LIM domain at its C-terminus. A missense mutation close to the LIM domain in *chs3-2D* leads to extreme dwarfism and activation of defense responses (Bi et al., 2011). It is likely that mutations in *slh1* and *chs3-2D* are sensed by the TIR-NB-LRR part of the protein, which triggers immune activation.

A second gain-of-function allele of *CHS3, chs3-1*, gives rise to a truncated CHS3 without part of the LIM domain (Yang et al., 2010). The phenotype of *chs3-1* is temperature dependent and manifested when grown at 16°C or lower. Another chilling sensitive mutant, *chs2-1*, also contains a gain-of-function mutation in a TIR-NB-LRR protein (Huang et al., 2010). A single amino acid substitution in the NB domain of RECOGNITION OF PERONOSPORA PARASITICA 4 (RPP4) causes constitutive defense activation when the mutant is grown at 16°C or lower. *CHS1* encodes a truncated TIR-NB protein. A missense mutation in CHS1 results in activation of cell death and defense responses at low temperature (Wang et al., 2013; Zbierzak et al., 2013).

*uni-1D* is the only known mutant of a CC-NB-LRR protein that causes autoimmunity (Igari et al., 2008). It carries three amino acid substitutions in the LRR domain. In addition to constitutive defense activation, *uni-1D* has a variety of developmental defects associated with increased cytokinin accumulation. It is unclear how activation of UNI leads to modifications in the cytokinin pathway.

## Autoimmunity Caused By Mutations in RLKS/RLPS

*Arabidopsis* Brassinosteroid Insensitive 1-associated receptor kinase 1 (BAK1) is a critical component of PTI which functions as co-receptor of multiple PRRs (Liebrand et al., 2014). Interestingly, knocking out both *BAK1* and its close homolog of *BAK1-like 1* (*BKK1*) leads to strong autoimmune phenotypes (He et al., 2007). Defense responses are not constitutively activated in the *bak1* and *bkk1* single mutants, but uncontrolled spreading of necrosis occurs in *bak1* knockout mutant plants upon infection by necrotrophic pathogens (Kemmerling et al., 2007). The mechanism of how loss-of-function of BAK1 and BKK1 activates plant immunity remains to be determined.

Several members of the BAK1-interacting RLK (BIR) subfamily have been shown to associate with BAK1 *in vivo* and serve as regulators of immunity (Gao et al., 2009; Halter et al., 2014). The *bir1-1* knockout mutant displayed extreme dwarfism, spontaneous cell death and constitutive defense responses (Gao et al., 2009). Loss of BIR2 function leads to enhanced PTI responses, but does not cause autoimmunity (Halter et al., 2014).

Gain-of-function mutations in two RLKs were shown to cause autoimmunity. In *snc4-1D*, a single amino acid substitution in the kinase domain of SNC4 leads to extreme dwarfism and constitutive defense responses (Bi et al., 2010). CHITIN ELICITOR RECEPTOR KINASE (CERK) 1 is an RLK involved in perception of chitin and bacterial peptidoglycan (Miya et al., 2007; Wan et al., 2008). In *cerk1-4*, a single amino acid change in the CERK1 ectodomain results in deregulated cell death upon pathogen challenge (Petutschnig et al., 2014).

*snc2-1D* is the only reported gain-of-function RLP mutant which causes autoimmunity in *Arabidopsis* (Zhang et al., 2010). Loss of function of SNC2 results in enhanced susceptibility to the non-pathogenic bacterium *P.s.t.* DC3000 *hrc*C, suggesting that SNC2 plays an important role in PTI. The alteration of a G to R in a conserved motif of the transmembrane helix in snc2-1D may disrupt the interaction between SNC2 and its negative regulator.

## Autoimmunity Caused By Mutations in Other Types of Protein

Mutations in a large number of genes encoding proteins not in the NB-LRR or RLK/RLP families were also found to result in autoimmunity. In some cases, the autoimmune phenotypes are caused by loss-of-function of negative regulators or gain-of-function of positive regulators of plant immunity. But most often, they are due to activation of immunity mediated by specific NB-LRR immune receptors, probably as targets of pathogen effectors that are monitored or guarded by Resistance proteins. One well-studied example is RPM1 INTERACTING PROTEIN (RIN) 4, which is guarded by two NB-LRR proteins RESISTANT TO P. SYRINGAE (RPS) 2 and RESISTANCE TO P. SYRINGAE PV MACULICOLA (RPM) 1 (Axtell and Staskawicz, 2003; Mackey et al., 2003). Loss of RIN4 triggers defense activation mediated by RPS2 and RPM1 (Belkhadir et al., 2004).

Loss-of-function mutations in several genes cause activation of SNC1-mediated immune responses. *CONSTITUTIVE EXPRESSER OF PR GENES* (*CPR*) 1 encodes an F-box protein that regulates the turnover of SNC1 (Cheng et al., 2011; Gou et al., 2012). Mutations in CPR1 cause elevated SNC1 and RPS2 protein levels and activation of defense responses. SUPPRESSOR OF RPS4-RLD (SRFR) 1 is also involved in regulating SNC1 protein levels. Mutations in SRFR1 result in increased SNC1 level and activation of SNC1-mediated immunity (Kim et al., 2010; Li et al., 2010a). Knockout mutants of *BONZAI* (*BON*) *1* and *MITOGEN-ACTIVATED PROTEIN KINASE PHOSPHATASE* (*MKP*) *1* also exhibit autoimmune phenotypes that are dependent on SNC1 (Yang and Hua, 2004; Bartels et al., 2009). How loss of BON1 and MKP1 leads to activation of SNC1-mediated immunity remains to be determined.

The autoimmune phenotypes of two lesion-mimic mutants, *lesion simulating disease* (*lsd*) *1* and *accelerated cell death* (*acd*) *11*, are also caused by activation of NB-LRR protein mediated immunity. The spontaneous cell death phenotype in *lsd1* is dependent on ACTIVATED DISEASE RESISTANCE (ADR) 1, ADR1-L1, and ADR1-L2, which function as “helper NB-LRRs” that assist other NB-LRR proteins to transduce defense signals (Bonardi et al., 2011). LAZARUS (LAZ) 5, a TIR-NB-LRR protein, is required for the cell death and constitutive defense phenotype in *acd11* (Palma et al., 2010). The constitutive defense response in another lesion-mimic mutant, *cpr5*, was also shown to associate with activation of NB-LRR protein mediated immunity (Wang et al., 2014).

*Arabidopsis* MAPK/ERK KINASE KINASE (MEKK) 1, MAP KINASE KINASE (MKK) 1/MKK2, and MAP KINASE (MPK) 4 form a MAPK cascade that plays critical roles in plant immunity (Gao et al., 2008; Qiu et al., 2008). Loss-of-function of MEKK1, MKK1/MKK2, or MPK4 leads to activation of defense responses (Ichimura et al., 2006; Nakagami et al., 2006; Suarez-Rodriguez et al., 2007). The autoimmune phenotypes of *mekk1, mkk1 mkk2*, and *mpk4* mutants are dependent on the CC-NB-LRR protein SUMM2, suggesting that the MEKK1-MKK1/MKK2-MPK4 cascade is monitored by SUMM2, which can sense its disruption by pathogen effectors (Zhang et al., 2012).

ACD6 and BIAN DA (BDA) 1 are two structurally related transmembrane proteins with an N-terminal ankyrin-repeat domain (Lu et al., 2003; Yang et al., 2012). *acd6-1* and *bda1-17D* are both gain-of-function mutations located in the transmembrane regions, which cause spontaneous cell death and constitutive defense responses. ACD6 positively regulates the abundance of several PRRs (Tateda et al., 2015). It likely functions as a critical regulator of PTI. BDA1 is required for the autoimmunity of *snc2-1D*, suggesting that it is a positive regulator of SNC2-mediated defense responses (Yang et al., 2012).

Mutations in multiple genes in the cyclic nucleotide-gated ion channel (CNGC) family have been shown to cause autoimmune phenotypes, highlighting the potential importance of calcium channels in plant immune regulation. Loss-of-function mutations in DEFENSE NO DEATH (DND) 1 (CNGC2) and DND2 (CNGC4) results in constitutive defense responses in the absence of cell death (Clough et al., 2000; Balagué et al., 2003; Jurkowski et al., 2004), suggesting that they play critical roles in negative regulation of plant defense. The spontaneous cell death and constitutive defense responses in the semi-dominant mutant *cpr22* are caused by a novel chimeric protein generated by a deletion that fused *CNGC11* and *CNGC12* (Yoshioka et al., 2006). Knockout analysis showed that both CNGC11 and CNGC12 function in positive regulation of pathogen resistance (Yoshioka et al., 2006).

Mutations in several other negative regulators of plant immunity were shown to cause activation of defense responses. Double knockout mutants of *NON-EXPRESSER OF PR GENES* (*NPR*) *3* and *NPR4* exhibit constitutive defense gene expression and enhanced resistance to pathogens (Zhang et al., 2006). T-DNA insertion mutants of *CALMODULIN-BINDING TRANSCRIPTION ACTIVATOR* (*CAMTA*) *3* displayed enhanced disease resistance and dwarfism when grown at low temperatures (Du et al., 2009), whereas a gain-of-function mutation in CAMTA3 leads to compromised immune responses (Jing et al., 2011; Nie et al., 2012). PLANT U-BOX (PUB) 13 is a E3 ubiquitin ligase involved in degradation of FLAGELLIN-SENSITIVE2 and the ABA co-receptor ABI1 (Lu et al., 2011; Kong et al., 2015). Knockout mutant plants of *pub13* exhibit spontaneous cell death and enhanced pathogen resistance (Li et al., 2012).

In some autoimmune mutants, the mechanism of how defense responses are constitutively activated is unclear. *cpr6-1* is a semi-dominant mutant with constitutive defense responses due to a mutation of unknown nature (Clarke et al., 1998). Loss-of-function of the stearoyl-ACP desaturase SSI2 activates NPR1-independent defense responses (Shah et al., 2001; Kachroo A. et al., 2003). The link between altered fatty acid metabolism and activation of immunity remains to be determined. Double knockout mutant plants of SYNTAXIN OF PLANTS (SYP) 121 and SYP122 display a lesion-mimic phenotype and enhanced pathogen resistance (Zhang et al., 2007). Loss-of-function mutations in two membrane attack complex/perforin proteins, CONSTITUTIVELY ACTIVATED CELL DEATH (CAD) 1 and NECROTIC SPOTTED LESIONS (NSL) 1, result in spontaneous cell death and constitutive defense response (Morita-Yamamuro et al., 2005; Noutoshi et al., 2006). The exact roles of CAD1 and NSL1 in immunity are unclear.

## Applications of Autoimmune Mutants in Genetic Analysis of Plant Immunity

One obvious feature of autoimmune mutants is the distinct morphological phenotypes such as dwarfism and spontaneous cell death. They are often used in epistasis analysis to test genetic interactions. Because large populations of plants can be screened using these morphological phenotypes as readout, a number of suppressor mutant screens have been carried out on various autoimmune mutants (**Table 2**). Mutants obtained from a suppressor screen can be either intragenic or extragenic suppressors. Intragenic suppressor mutations can be useful for structure-function analysis, whereas extragenic suppressors are valuable for identifying components required for the autoimmune phenotypes in the original mutant.

**Table 2:** Suppressor screens carried out on known *Arabidopsis* autoimmune mutants.

Mutant	Suppressors identified	Reference
*mkk1 mkk2*	*summ* mutants	Kong et al., 2012
*acd11*	*laz* mutants	Palma et al., 2010
*lsd1*	*phx/adr* mutants	Bonardi et al., 2011
*bir1*	*sobir* mutants	Gao et al., 2009
*snc1*	*mos* mutants	Zhang and Li, 2005
*snc4-1D*	6 intragenic, *sua, rsn2*	Zhang et al., 2014
*snc2-1D*	6 intragenic, *bda* mutants	Zhang et al., 2010
*ssi2*	*rdc* and *sfd* mutants	Kachroo P. et al., 2003
*acd6-1*	17 intragenic, *sup6*	Lu et al., 2009
*slh1*	*rps4*, mutants, 14 intragenic	Sohn et al., 2014
*syp121 syp122*	*fmol, aldl, pad4*	Zhang et al., 2008
*chs2*	1 intragenic, *sgt1b, rar1*	Huang et al., 2010
*cpr22*	Intragenic suppressors	Baxter et al., 2008
*bak1 bkk1*	*sbb1, stt3a*	Du et al., 2016;
		de Oliveira et al., 2016

One very useful feature of autoimmune mutants is that the dwarf phenotype is often dependent on environmental conditions such as temperature and humidity. For example, the dwarf phenotype of *bon1* and *snc4-1D* can be fully suppressed and the dwarf phenotype of *snc2-1D, mkk1 mkk2*, and *bir1-1* can be partially suppressed when grown at 28°C (Hua et al., 2001; Gao et al., 2009; Bi et al., 2010; Zhang et al., 2010; Zhang et al., 2012). The dwarf phenotype of several other mutants such as *ssi4, cpr22*, and *slh1* can be suppressed by high humidity (Yoshioka et al., 2001; Zhou et al., 2004; Noutoshi et al., 2005). Temperature and humidity sensitivity are frequently observed in the same mutants, suggesting a common element of these phenotypes, although the molecular mechanisms behind these conditional phenotypes are not clear yet. For mutants with extreme dwarf morphology, seeds of a mutagenized mutant population can be obtained by growing plants at high temperature or high humidity. Mutant screens can be subsequently carried out in the next generation at regular growth conditions. The conditional features of the autoimmune mutants allow screening of large mutagenized populations, enabling systematic discovery.

Analysis of suppressors of autoimmune mutants has been very effective for investigating the molecular basis of the respective constitutive defense responses. For example, studies on *suppressor of mkk1 mkk* (*summ*) *2* revealed that the autoimmune phenotypes in *mekk1, mkk1 mkk2*, and *mpk4* are due to activation of immune responses mediated by the CC-NB-LRR protein SUMM2 (Zhang et al., 2012). Characterization of suppressor mutants of *acd11* revealed that loss of ACD11 results in activation of immune responses mediated by the TIR-NB-LRR protein LAZ5 (Palma et al., 2010). Analysis of suppressor mutants of *lsd1* revealed that ADR1, ADR1-L1, and ADR1-L2 are required for the lesion mimic phenotype of *lsd1* (Bonardi et al., 2011), suggesting that NB-LRR-mediated defense responses are activated in the mutant. Studies on suppressors of *bir1-1* revealed that loss of BIR1 leads to activation of immune responses mediated by RLKs BAK1 and SUPPRESSOR OF BIR1 (SOBIR) 1 (Gao et al., 2009; Liu et al., 2016).

Suppressors of autoimmune mutants have been very useful for studying the biogenesis of plant immune receptors and dissecting signaling pathways downstream of plant immune receptors. For example, analysis of suppressors of *snc1* revealed that *SNC1* is regulated at both transcriptional and post-transcriptional levels and highlights the importance of nucleocytoplasmic trafficking and RNA processing in plant immunity (Palma et al., 2005, 2007; Zhang and Li, 2005; Zhang et al., 2005; Cheng et al., 2009; Germain et al., 2010; Li et al., 2010b; Xu et al., 2011, 2012; Xia et al., 2013). The identification of MODIFIER OF SNC1 (MOS) 5 suggests that protein ubiquitination plays an important role in immunity mediated by NB-LRR proteins (Goritschnig et al., 2007). From the *snc1* suppressor screen, the transcriptional repressor TOPLESS RELATED 1 was identified as a critical plant immune regulator that suppresses the expression of negative regulators of immunity (Zhu et al., 2010). Identification of *set domain group 8* (*sdg8*) as a suppressor of *acd11* revealed that the expression of *LAZ5* is regulated by histone modifications (Palma et al., 2010). Studies on *snc4-1D* suppressor mutants revealed critical roles of alternative splicing in immunity mediated by the RLKs SNC4 and CERK1 (Zhang et al., 2014).

Analysis of suppressor mutants of *snc2-1D* identified BDA1 as a critical regulator of PTI (Yang et al., 2012). It also identified WRKY DNA-BINDING PROTEIN (WRKY) 70 as a critical regulator of the SA-independent defense pathway (Zhang et al., 2010). The identification of *gtp binding protein beta* (*agb*) *1* as a suppressor of *bir1-1* and subsequent studies revealed that heterotrimeric G-proteins serve as a converging point for immunity activated by multiple RLKs (Liu et al., 2013).

In addition to suppressor screens, autoimmune mutants can also be used for enhancer screens. For example, a genetic screen looking for enhancers of *snc1* identified many *muse* (*mutants, snc1-enhancing*) mutants (Huang et al., 2013). Analysis of the *muse* mutants lead to the identification of several components involved in proteasome-mediated degradation of SNC1 (Huang S. et al., 2014; Huang Y. et al., 2014; Huang et al., 2016). It also revealed a crucial role of N-terminal acetylation on the turnover of SNC1 (Xu et al., 2015).

## Concluding Remarks

Mutant analyses have been instrumental in the mechanistic studies of plant immunity. A wide range of autoimmune mutants has been identified in *Arabidopsis*. In some of these mutants, the mechanism of defense activation is unclear. Interpretation of data obtained from them is often difficult, especially when the mutations occur in proteins involved in general biological processes. However, the autoimmune phenotypes of many mutants are caused by activation of defense responses mediated by specific plant immune receptors. Their distinct phenotypes facilitate easy and fast identification of suppressor or enhancer mutants, enabling new discoveries and allowing researchers to study signaling mechanisms in plant immunity.

## Author Contributions

All authors listed, have made substantial, direct and intellectual contribution to the work, and approved it for publication.

## Conflict of Interest Statement

The authors declare that the research was conducted in the absence of any commercial or financial relationships that could be construed as a potential conflict of interest.
